# Detection of cytogenetically cryptic *PML-RARA* fusion in acute promyelocytic leukemia by rapid next generation sequencing

**DOI:** 10.1038/s41698-025-01072-8

**Published:** 2025-08-09

**Authors:** Hailee St. Louis, Nicholas Protopsaltis, Erik Ames, Tiffany Tanaka, Wei Song

**Affiliations:** 1 Department of Pathology, University of California San Diego, La Jolla, CA USA; 2 Department of Pathology, Stanford Medicine, Stanford University, Stanford, CA USA; 3 Department of Medicine, University of California San Diego, La Jolla, CA USA

**Keywords:** Biomarkers, Oncology, Pathogenesis

## Abstract

Acute promyelocytic leukemia (APL) is a medical emergency that requires treatment to be initiated as soon as the disease is suspected in order to decrease the risk of early death, which is mostly caused by APL-associated coagulopathy. Currently, the diagnosis of APL relies on the detection of *PML-RARA* fusion by fluorescence in situ hybridization (FISH). However, rare cases are cytogenetically cryptic, negative by FISH, and require other molecular techniques to detect the fusion. Moreover, next generation sequencing (NGS) has not been incorporated in the routine diagnosis of leukemia-associated fusions. Hereby, we present a clinical APL case, reported negative by FISH, where *PML-RARA* fusion was detected by next generation sequencing (NGS) within 48 h, indicating the benefit of incorporating rapid NGS into the routine diagnostic management of acute leukemia patients.

## Introduction

Acute promyelocytic leukemia (APL) is a type of acute myeloid leukemia (AML) characterized by abnormal promyelocytes and the *PML-RARA* fusion^1^. It accounts for approximately 10–15% of newly diagnosed AML cases^2^. APL presents with abnormal blood counts and coagulopathy largely driven by disseminated intravascular coagulopathy (DIC)^1,3^. Historically, APL was highly fatal due to the rapid progression of disease with severe coagulopathy leading to hemorrhagic complications. The discovery of t(15;17) and use of the differentiating agent all-trans retinoic acid (ATRA) has transformed APL into the most curable subtype of adult AML. At present, ATRA-based regimens result in complete remission in nearly all cases^2^. Although multicenter clinical trials have reported an early death rate of 5–10%, population-based studies have shown a much higher rate of early death from disease. In a Swedish study, 13% of early patient deaths occurred before treatment was started, and 30% occurred on the day of diagnosis or the day after^4,5^. Although APL may be highly curable, the high risk of early death illustrates the critical urgency of rapid diagnosis.

The defining feature of APL is the balanced chromosomal translocation t(15;17)(q24;q21) leading to fusion of the promyelocytic leukemia (*PML*) gene with the retinoic acid receptor alpha (*RARA*) gene. There are three *PML-RARA* transcript isoforms that have been identified: (i) long isoform (breakpoint cluster region 1, bcr-1) due to a breakpoint in *PML* (intron 6), (ii) variant isoform (breakpoint cluster region 2, bcr-2) due to a breakpoint in *PML* (exon 6), and (iii) short isoform (breakpoint cluster region 3, bcr-3) due to a breakpoint in *PML* (intron 3)^1,6^. In addition, there are a subset of cases with variant translocations involving *RARA*, including t(11;17)(q23;q21) that produces the *PLZF-RARA* fusion, t(5;17)(q35;q21) that forms *NPM1-RARA*, t(11;17)(q13;q21) that generates *NUMA-RARA*, and der(17) that creates *STAT5B-RARA*^7^. These are extremely important to identify as some variant translocations respond poorly to ATRA and arsenic trioxide (ATO) therapies.

In the majority of cases, the fusion is detected by conventional karyotype and fluorescence in situ hybridization (FISH). However, there are rare, cytogenetically cryptic, cases of APL that lack detectable *PML-RARA* fusion by conventional chromosomal analysis or FISH. These cases make up approximately less than 1% of APL cases^3,8^. The most prevalent confirmatory technique in these cases is RT-PCR, which is also capable of identifying isoforms but cannot identify variant translocations involving *RARA*^9^.

In this report, we present a case of cytogenetically cryptic APL with suspicious morphologic and typical clinical findings, where the t(15;17)/*PML-RARA* fusion was not identified by conventional cytogenetic analysis or FISH. However, the fusion was detected by a myeloid-directed rapid next generation sequencing (NGS) panel prior to confirmation by RT-PCR in a very short period.

## Results

### Patient clinical presentation and cytogenetics finding

**Fig. 1:**
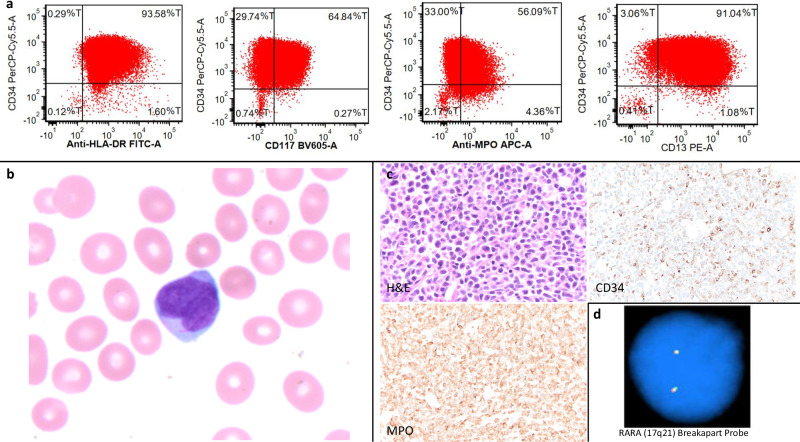
Histology, flow cytometry, immunohistochemistry and FISH results. **a** Flow cytometry showed CD34 (+) and HLA-DR (+) which is not common in hypergranular acute promyelocytic leukemia; however, CD34 positivity can be seen in microgranular acute promyelocytic leukemia which is more consistent with this patient’s presentation. **b** Microgranular promyelocyte in peripheral blood (wright Giemsa stain). **c** Bone marrow core. **d** Negative RARA break apart probe set (Abbott Molecular, Inc.) to detect gene rearrangement at 17q12-q21 associated with acute promyelocytic leukemia (APL).

A 51-year-old female presented to an outside hospital with bruising and headache for 3 days. Initial CBC showed elevated white blood cell (WBC) 20,540 /mm^3^, hemoglobin (Hgb) 6.8 gm/dL, platelets (PLT) 17,000 /mm^3^, and peripheral smear with leukocytosis and atypical blasts. The patient was transferred to our institution due to concern for acute leukemia. Her labs also indicated disseminated intravascular coagulation (DIC) with a PT 18.2 s, PTT 26 s, fibrinogen 66 mg/dL, and D-dimer 1,667 ng/mL. On peripheral smear review at our institution, atypical blasts were noted with cleaved nuclei and rare Auer rods. Morphologically, the blasts were highly suspicious for APL and the patient was empirically started on ATRA therapy. Flow cytometry of the peripheral blood revealed 95% abnormal myeloid blasts, which were positive for CD13, CD33, CD64, CD117 (major subset), and MPO (Fig. 1). In addition, they were also notably positive for CD34 and HLA-DR. While CD34 is more frequently expressed in the microgranular variant, HLA-DR expression is uncommon in APL. The bone marrow biopsy showed sheets of blasts that were CD117 and MPO diffusely positive by immunohistochemistry, with CD34 showing variable reactivity. Chromosome analysis showed a normal female karyotype with no abnormalities detected by an AML FISH panel, including *RARA* break apart analysis (Fig. 1).

### NGS detected PML-*RARA* fusion and confirmed by RT-PCR

**Fig. 2:**
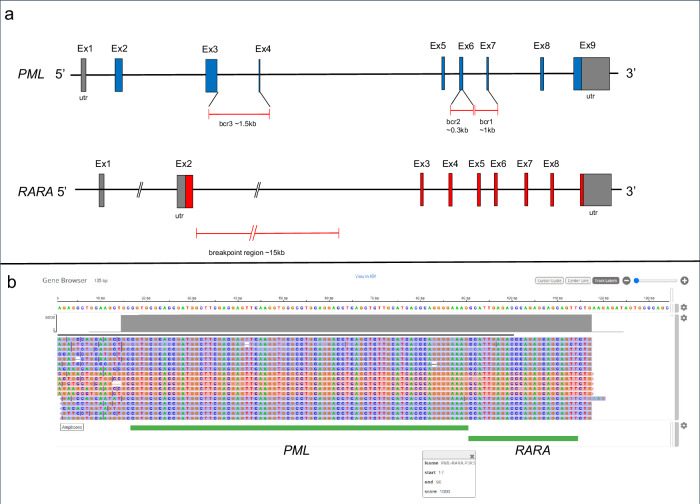
PML-RARA fusion detection by NGS. **a** Schematic representation of PML and RARA genes as well as the breakpoint locations for different isoforms. different gene products of the PML-RARA isoforms. **b** IGV view of PML-RARA fusion by NGS-based RNAseq detection.

Concurrently, our institution routinely performs a rapid NGS myeloid panel, interrogating 45 DNA target genes and 34 RNA fusion driver genes, (Oncomine Myeloid Assay GX v2, Thermo Fisher Scientific) for all newly diagnosed leukemia patients with an approximately 48-hour turn-around time. For this patient, the rapid NGS panel detected a *PML*(3)-*RARA*(3) fusion and FLT3-ITD 48 h after the sample arrived to the lab (Fig. 2). At this time ATRA with ATO was initiated, followed by discontinuation of ATO and initiation of idarubicin for rapid cytoreduction due to rise in white blood cell count >50,000 /mm^3^ and subsequent intubation for hypoxemic respiratory failure from differentiation syndrome. A RT-PCR test was sent to an outside central laboratory and the result showed the *PML-RARA* short fusion transcript associated with t(15;17)(q22;q21), confirming the results from the our rapid NGS panel ~16 days later. APL with bcr3 short isoform often displays microgranular type blasts consistent with this patient’s presentation. A follow up bone marrow biopsy one month after starting induction treatment showed a complete remission, with no morphologic or immunophenotypic evidence of APL, and FLT3-ITD mutation was not detected by PCR. She stabilized and was discharged to home 46 days after her initial presentation, and is currently completing consolidation therapy with ATRA + ATO. Incorporating the molecular profiling results, including NGS, RT-PCR, cytogenetics and FISH, this case represents a cryptic *PML-RARA* fusion with FLT3 ITD.

## Discussion

Cytogenetically cryptic APL is a rare phenomenon that occurs in approximately less than 1% of APL cases and has been shown to occur with all three *PML-RARA* transcript isoforms^3,8,9^. Although FISH for *RARA* rearrangement commonly represents the gold-standard for APL detection, flow cytometry is often employed as an additional screen to “rule out” APL in the critical initial hours/days after presentation. This evaluation is often based on the characteristic immunophenotype of myeloid blasts lacking CD34 and HLA-DR. Interestingly, a study of 132 cases of APL showed this immunophenotype was only present in 74% of APL blasts and frequently seen in non-APL AML^10^. In our case, the lack of a characteristic immunophenotype was compounded by cryptic cytogenetics and illustrates the benefit of utilizing complementary techniques, as early diagnosis and initiation of treatment dramatically improve outcomes in APL.

NGS-based genomic profiling has been widely adapted in the routine care of cancer patients, specifically for disease diagnosis, risk stratification, treatment selection and disease surveillance^11^. For example, RNAseq-based fusion detection has been widely used in the detection of translocations which are targetable by some tyrosine kinase inhibitors, such as ALK fusion in non-small cell lung cancer (NSCLC) patients^12,13^. However, this technique has not been routinely incorporated into the fusion detection of hematopoietic neoplasms which still mainly relies on FISH. Compared to break apart FISH, NGS-based fusion detection has the following advantages: 1) NGS can detect specific partner genes in fusions, which carry different clinical significance. For example, APL patients could carry *PLZF-RARA*, *NPM1-RARA*, *NUMA-RARA*, and *STAT5B-RARA*, although the majority are caused by *PML-RARA* fusion. However, except for *PML-RARA*, patients with other fusion types respond poorly to ATRA-based therapies. 2) NGS covers different types of alterations, and therefore conveys a more comprehensive genomic profiling of each cancer patient. In our case, we also detected *FLT3* ITD in addition to *PML-RARA*. This all-in-one advantage also reduces the specimen requirements and turn*-*around time needed for multiple individual assays.

Since the introduction of comprehensive NGS testing into the frontline of patient care almost 10 years ago^11^, the advances in workflow automation have significantly accelerated the speed from sample collection to report. The turn-around time of clinical NGS testing has been shortened from average 14 days to 1–2 days^14,15^. In our case, the patient presented with a strong clinical suspicion of APL due to cytopenias, leukocytosis with characteristic blasts, and DIC. However, initial flow cytometry had findings not commonly seen in APL, with FISH negative for *PML-RARA* fusion. The Rapid NGS testing was the first method to detect the fusion within 2 days. Therefore, incorporating fast turn-around-time NGS testing into acute leukemia patient care empowers clinicians to make rapid biomarker-informed decision and improved outcomes.

## Methods

### Consent

The patient has consented to the publication of this report verbally. This study was approved by the Institutional Review Boards at University of California San Diego (IRB # 808807). All handling of patient data was performed in accordance with the Declaration of Helsinki.

### FISH test

RARA break apart probe set (Abbott Molecular, Inc.) was used to detect gene rearrangement at 17q12-q21 associated with acute promyelocytic leukemia (APL).

### NGS test

NGS testing was performed on the Genexus Integrated Sequencer (Thermo Fisher Scientific) using the Oncomine Myeloid Assay GX v2 (Thermo Fisher Scientific) targeting 540 DNA and 779 RNA amplicons spreading across 45 genes and 34 fusion driver genes.

## Data Availability

No datasets were generated or analysed during the current study.
